# Supplementary material for: Refractive error among school-aged children in Bhutan: A secondary analysis of data from nationwide Bhutan School Sight Survey
Source: PLOS Glob Public Health. 2026 Feb 17;6(2):e0005085. doi: 10.1371/journal.pgph.0005085 (PMC12912612; doi:10.1371/journal.pgph.0005085)
Supplement: S1 Table — (DOCX) [file pgph.0005085.s001.docx]

**S1 Table:** Prevalence of refractive errors among students in all 20 districts (Dzongkhags) and four urban towns (Thromde) in Bhutan.

| **Sl #** | **Dzongkhag/Thromde** | **# of Schools screened (%)** | **Total No. of students Screened** | | | **Total No. of Refractive Errors** | | | **Total % of RE** |
| --- | --- | --- | --- | --- | --- | --- | --- | --- | --- |
|  |  |  | Male | Female | Total | Male | Female | Total |  |
| 1 | Bumthang | 19 (100) | 2093 | 2130 | 4223 | 178 | 230 | 408 | 9.66 |
| 2 | Chukha | 42 (100) | 5020 | 5207 | 10227 | 463 | 539 | 1002 | 9.8 |
| 3 | PhuentsholingThromde | 6 (100) | 2344 | 2302 | 4646 | 300 | 401 | 701 | 15.09 |
| 4 | Dagana | 24 (100) | 3108 | 3185 | 6293 | 252 | 315 | 567 | 9.01 |
| 5 | Gasa | 6 (76) | 420 | 366 | 786 | 45 | 61 | 106 | 13.49 |
| 6 | Haa | 11 (100) | 1769 | 1777 | 3546 | 196 | 259 | 455 | 12.83 |
| 7 | Lhuentse | 19 (100) | 1678 | 1807 | 3485 | 131 | 158 | 289 | 8.29 |
| 8 | Mongar | 49 (100) | 4610 | 4899 | 9509 | 340 | 476 | 826 | 8.69 |
| 9 | Paro | 30 (100) | 5474 | 5723 | 11197 | 540 | 836 | 1356 | 12.11 |
| 10 | P/Gatsel | 25 (100) | 2775 | 2866 | 5641 | 211 | 289 | 504 | 8.93 |
| 11 | Punakha | 26 (100) | 3354 | 3629 | 6983 | 346 | 430 | 776 | 11.11 |
| 12 | Samdrup Jongkhar | 27 (100) | 2837 | 3056 | 5893 | 220 | 270 | 490 | 8.31 |
| 13 | SJ Thromde | 5 (100) | 1024 | 1074 | 2098 | 130 | 149 | 279 | 13.3 |
| 14 | Samtse | 46 (100) | 7351 | 7111 | 14462 | 508 | 626 | 1134 | 7.84 |
| 15 | Sarpang | 22 (100) | 4040 | 4184 | 8224 | 580 | 687 | 1277 | 15.53 |
| 16 | Gelephu Thromde | 4 (100) | 1520 | 1698 | 3218 | 268 | 330 | 598 | 18.58 |
| 17 | Thimphu | 13 (100) | 1754 | 1779 | 3533 | 232 | 265 | 497 | 14.07 |
| 18 | Thimphu Thromde | 34 (97.1) | 11171 | 11757 | 22928 | 1867 | 2179 | 4047 | 17.65 |
| 19 | Tashigang | 56 (100 | 5199 | 5681 | 10880 | 529 | 701 | 1223 | 11.24 |
| 20 | T/Yangtse | 28 (100) | 2269 | 2387 | 4656 | 196 | 256 | 452 | 9.71 |
| 21 | Trongsa | 19 (100) | 1685 | 1885 | 3570 | 144 | 201 | 345 | 9.66 |
| 22 | Tsirang | 16 (100) | 2767 | 2728 | 5495 | 274 | 299 | 573 | 10.43 |
| 23 | Wangdiphodrang | 34 (100) | 4014 | 4360 | 8374 | 418 | 500 | 918 | 10.96 |
| 24 | Zhemgang | 28 (100) | 2193 | 2305 | 4498 | 195 | 223 | 418 | 9.29 |
|  | GRAND TOTAL | 586 (99.3) | 80469 | 83896 | 164365 | 8563 | 10680 | 19241 |  |
