# Supplementary material for: Refractive error among school-aged children in Bhutan: A secondary analysis of data from nationwide Bhutan School Sight Survey
Source: PLOS Glob Public Health. 2026 Feb 17;6(2):e0005085. doi: 10.1371/journal.pgph.0005085 (PMC12912612; doi:10.1371/journal.pgph.0005085)
Supplement: Attachment — Submitted filename: Response to reviewers.docx [file pgph.0005085.s006.docx]

Refractive error among school-aged children in Bhutan: A secondary analysis of data from nationwide Bhutan School Sight Survey

**Submission ID: PGPH-D-25-02130**

**Response to Reviewer #1**

We sincerely thank the reviewer for the review and constructive comments that helped improve our manuscript. We have carefully considered all the points raised, incorporated changes and revised the manuscript to improve clarity, consistency, and structure. We have also corrected a few grammatical errors overlooked in our previous submission.

| **Sl no** | **Section** | **Comments** | **Authors Response** |
| --- | --- | --- | --- |
| 2. | Abstract | Is the rate different statistically? If it is, please report the p value. | Thank you for the comments. Yes, there is a statistically significant difference. We have added the p values. Additionally, we have added the Adjusted OR values. |
| 3. | Introduction | Rephrase: Consider ‘increase in URE’. | The sentence is rephrased as suggested. |
| 4. | Introduction | Is this challenge only impacting delivery of paediatric eye care? Does it not impact eye care generally? I feel that a broader concept should be addressed notwithstanding that the focus of the current paper is on URE on school-aged children. | Thank you for this suggestion. We agree that the challenge of mountainous geography and widely dispersed rural population impacts general eye care. We have rephrased the sentence accordingly. |
| 5. | Methodology (Ethical consideration) | Ideally, I would imagine that the parents of the children will be the one to give consent for their children to be examined. School authorities can only give approval for the exercise to be conducted in their school. Could the authors explain why consent was obtained from school authorities rather than parents. | Thank you for raising this point.  We are providing the following justification in the manuscript.  ‘Ethical approval for this study was obtained from the Research Ethics Board of Health (REBH), and administrative clearance from the Policy and Planning Division, Ministry of Health, Bhutan. The REBH also approved this secondary analysis of anonymized data without requiring re-consent.  The BSSS survey received ethical approval from the Research Ethics Board of Health (REBH), Ministry of Health, Bhutan, with additional authorization granted by the Ministry of Education. BSSS was implemented by the Ministry of Health as a public health intervention for school children using standardized eye examination protocols rather than a research study involving personal risk or sensitive procedures.  In Bhutan, school health activities such as medical or vision screening are considered part of the routine school health program, conducted under the joint authority of the Ministry of Health and the Ministry of Education. Written informed consent was obtained from school authorities, including principals and teachers on behalf of students, as they act *in loco parentis* (in the place of parents) during school hours. Moreover, verbal assent was obtained from all participating students to ensure voluntary participation. Since the procedures involved only non-invasive eye examinations and free spectacle provision, the REBH deemed parental written consent unnecessary, provided that school authorities had granted approval and that ethical oversight was maintained throughout. |
| 6. | Results | Please report the p-value for the difference in proportion to show if there is indeed a statistical difference in the proportion of VI between male and female school children or not. | We had missed to report it. There is a statistical difference in the proportion of VI between male and female school children. We have now mentioned the p value. |
| 7. | Results | It is good practice to have tables on a single page if possible. | Thank you for the suggestion. We have now put it in a single page. However, we understand that formatting of tables would be considered during the production. |
| 8. | Results | Report percentage as well. | We have revised Table 3, with additional reporting of percentage and p -values. |
| 9. | Results | Reduce font size. | We have revised Tables to adjust font sizes. However, we understand that formatting of tables would be considered during the production. |
| 10. | Discussion | Given ref number 10, will it be accurate to describe this paper as one of the first that provides national prevalence of RE in school children in Bhutan? | Thank you for this comment.  We assumed that this paper (census data) estimated the first national prevalence of RE in Bhutan. However, to avoid confusion, we have rephrased the sentence. |
