# Supplementary material for: Refractive error among school-aged children in Bhutan: A secondary analysis of data from nationwide Bhutan School Sight Survey
Source: PLOS Glob Public Health. 2026 Feb 17;6(2):e0005085. doi: 10.1371/journal.pgph.0005085 (PMC12912612; doi:10.1371/journal.pgph.0005085)
Supplement: Attachment — Submitted filename: PGPH-D-25-02130_R1_review.docx [file pgph.0005085.s005.docx]

**Summary**

The revised version of the manuscript, “Refractive error among school-aged children in Bhutan: A secondary analysis of data from nationwide Bhutan School Sight Survey” has been improved in clarity, rigor, and presentation. The authors have provided a thorough and satisfactory revision. All substantive and other concerns raised in the initial review have been appropriately addressed.

**Evaluation**

The authors have responded to all reviewer comments in a systematic and constructive manner. Each point raised in the original review has been either directly addressed through revisions to the manuscript text, or adequately clarified with corresponding modifications that improve transparency and interpretability. In addition to addressing the specific reviewer comments, the authors have also corrected previously unforeseen grammatical errors throughout the manuscript. These improvements have enhanced readability and ensured a clearer communication of the study’s objectives, methods, results, and conclusions. The revisions do not introduce any new scientific concerns. The study remains methodologically sound, the analyses are appropriately described, and the conclusions are supported by the presented data.
